# Digital Access for Screens and Smiles: Telehealth Use and Dental Care Access in the U.S.

**DOI:** 10.3390/clinpract16050091

**Published:** 2026-05-06

**Authors:** Gulzar Shah, Anjana Kalathimekkattu, Indira Karibayeva, Bobbie J. Newell

**Affiliations:** Department of Health Policy and Community Health, Jiann-Ping Hsu College of Public Health, Georgia Southern University, Statesboro, GA 30460, USA; ak19676@georgiasouthern.edu (A.K.); ik01379@georgiasouthern.edu (I.K.); bnewell@georgiasouthern.edu (B.J.N.)

**Keywords:** teledentistry, telemedicine, dental care access, cost-related delay in care, oral health disparities, health services utilization, digital health, national health interview survey, oral health policy

## Abstract

**Background/Objectives:** Cost-related delays hinder access to dental care in the United States, while the growing use of telehealth has an uncertain impact on population-level access to dental services. The study examined factors associated with cost-related delays in dental care among U.S. adults and whether telemedicine use in the past year was associated with these delays. **Methods:** This cross-sectional study utilized nationally representative data from the 2023 National Health Interview Survey (NHIS) to examine the association between telehealth use and oral health services utilization in the past 12 months. All analyses were performed using R version 4.5.1 within the RStudio IDE (version 2025.09.0+387). **Results:** Adults who visited a dental provider within the past 12 months had lower odds of cost-related dental care delay, whereas telemedicine users had higher odds of delay. Higher odds of delayed dental care were observed among women, those aged below 65 years, Hispanics, those with poorer health, and those living in the Midwest, South, and West. Education and family income-to-poverty ratio were not significantly associated with delay in the adjusted analysis. **Conclusions:** Cost-related delays in dental care remain common and reflect persistent sociodemographic inequities and patterns of healthcare engagement. Telemedicine use alone does not appear to contribute to overcoming barriers to dental care access. Policies that reduce financial barriers and connect telehealth encounters with oral health referral pathways may improve equitable access to dental services.

## 1. Introduction

The oral health sector in the United States faces significant challenges that highlight the broad inequities in the health system. One of the key contributors to oral health disparities is socioeconomic factors [1]. In national surveys, a meaningful proportion of U.S. adults report delaying dental care because they cannot afford it. Such cost-related delay or lack of care may lead to poor oral health, pain, and avoidable use of urgent or emergency care services [2]. Poor oral health is also associated with poor overall health and well-being. Therefore, understanding which population subgroups experience cost-related delays in dental care remains a policy and practice priority for public health and health agencies in the United States [3]. In addition, social determinants such as low income, inadequate insurance, and low oral health literacy often lead to delay or neglect of dental care, contributing to malnutrition, reduced quality of life, and social isolation [4]. Vulnerable populations, including pregnant women, individuals with disabilities, Special Health Needs (SCHN), and underserved communities, face heightened oral health disparities that exemplify broader access barriers [5,6,7].

These patterns of unmet need across disadvantaged groups underscore the need for strategies to reduce barriers to dental care, particularly for individuals facing financial constraints. At the same time, the rapid expansion of telemedicine in the United States has changed how many patients interact with the health system [8]. Telemedicine use may serve as an indicator of broader healthcare engagement, digital access, and care-seeking behavior, which could be associated with whether individuals obtain timely preventive services, including dental care [9]. Emerging teledentistry applications (teleorthodontics, TMD management, OMFS) demonstrate feasibility for specific conditions but remain limited in population-level implementation [10,11,12,13,14]. This study examines whether general telemedicine use in the past 12 months is associated with cost-related delay in dental care among U.S. adults. In this study, the term telehealth describes remote health services; telemedicine refers to remote clinical care; and teledentistry refers to dental care delivered remotely [15]. Telemedicine use in the past 12 months, as measured in NHIS, is analyzed in this study, whereas teledentistry is discussed only as contextual literature and was not directly measured in this dataset. 

Teledentistry can aid providers in working as a collaborative team by enabling better inter-personal communication, more instantaneous and efficient. The increased interest in telehealth across dentistry underscores the necessity to ensure providers’ willingness and capability to adapt to such practices. It also calls for developing appropriate strategies to ensure the successful integration of telehealth services into dental practice. Telehealth utilization is influenced by a complex combination of demographic, socioeconomic, and systemic factors [16,17]. Research indicates that younger individuals, females, those with higher educational attainment, and individuals with private health insurance are more likely to participate in telehealth visits [8,16]. Factors such as having a regular source of care, access to the internet, and living in urban areas are also associated with higher rates of telehealth use. Conversely, individuals with lower incomes or limited digital literacy may face barriers to accessing telehealth services, potentially exacerbating existing disparities. These trends suggest that telehealth is not universally accessible and may replicate or even exacerbate current inequities in healthcare access, including dental care. Social determinants, pandemic disruptions, and regional workforce shortages further exacerbate cost-related dental care delays across diverse U.S. populations [18].

While telehealth has been widely studied in the contexts of mental health and chronic disease management, its potential to facilitate or replace access to oral healthcare remains underexplored. Some pilot studies on teledentistry indicate that it can be an effective triage tool, particularly in underserved communities or rural areas [19,20]. However, its adoption remains much less widespread than in other areas of healthcare. Furthermore, existing research has not adequately examined whether telehealth engagement correlates with subsequent dental service utilization, either directly or through referrals. Understanding the relationship between general telehealth engagement and dental care access could provide valuable insights into how digital health behaviors interact with broader patterns of preventive care-seeking.

Despite rapid growth in telemedicine use, there is limited national-level evidence on whether telemedicine engagement is associated with access to dental care, particularly cost-related delays in obtaining needed dental services [20]. Teledentistry feasibility has been discussed in the past literature. Still, only limited national evidence has examined, a priori based on theory, whether general telemedicine use is associated with cost-related delay in dental care among U.S. adults. Implementation of telehealth in a private pediatric dental clinic during the COVID-19 pandemic indicated that nearly half of emergencies could be managed via teledentistry [21].

To better frame our current analysis, we conceptualized telemedicine use as an indicator of broader healthcare engagement rather than as a direct measure of dental service integration. Cost-related dental care delays are recognized as the product of predisposing, enabling, and need-related factors that define healthcare access. This understanding is consistent with the Anderson Behavioral model, within which telemedicine may reflect patterns of care-seeking, digital access, and connection to the health system, while dental care access remains influenced by financial barriers, insurance coverage, referral pathways, and other structural constraints. Several important contextual factors, including digital literacy and detailed insurance coverage, are not fully captured in the NHIS and should be considered when interpreting results. This study utilizes 2023 National Health Interview Survey (NHIS) data to examine sociodemographic, economic, and health-related factors associated with cost-related dental care delays among U.S. adults.

In addition, the study sought to investigate whether the use of telemedicine services in the past 12 months was associated with cost-related delays in dental care.

## 2. Materials and Methods

### 2.1. Study Design and Data Source

This study utilized cross-sectional data from the 2023 NHIS, a population-based household health survey administered annually by the National Center for Health Statistics (NCHS), part of the U.S. Centers for Disease Control and Prevention (CDC). The NHIS employs a stratified multistage probability sampling design to ensure a representative sample of the civilian, noninstitutionalized U.S. population. The sampling frame is derived from the U.S. Census Bureau’s Master Address File and incorporates oversampling of minority populations to enhance statistical precision. Data collection for the 2023 NHIS was conducted from January through December 2023. The adult sample comprised individuals aged 18 years and older, selected randomly within each household. A total of 29,533 sample adults completed the survey. The conditional response rate for the adult interview was 89.6%, and the final response rate for the adult sample was 50.7%. All analyses adhered to the NHIS guidelines for complex survey design, applying sampling weights, strata, and primary sampling units to account for unequal probability of selection and non-response. The NHIS is publicly available and de-identified; therefore, this secondary analysis was determined not to constitute human subject research, and institutional review board (IRB) review was waived by the Georgia Southern University IRB (protocol #H26094, dated 22 October 2025).

### 2.2. Dependent and Independent Variables Description

The outcome variable was delay in dental care due to cost in the past 12 months, measured by the NHIS variable DENDL12M_A, which asks “During the past 12 months, have you DELAYED getting dental care because of the cost?” The original responses included “Yes,” “No,” “Refused,” “Not Ascertained,” and “Don’t Know.” For this analysis, the variable was recoded into a binary indicator: Yes = reported delay (coded as 1); No = no delay (coded as 0). Responses marked as “Refused,” “Not Ascertained,” or “Don’t Know” were excluded from the analytic sample.

The primary independent variable was recent dental care utilization, derived from DENPREV_A, which captures time since last dental exam or cleaning. This variable was recoded into a binary indicator: Yes = saw a dentist or dental provider within the past 12 months; No = all other response categories (longer than 12 months ago or never).

Additional covariates included: (1) Telemedicine use in the past 12 months (VIRAPP12M_A), dichotomized into Yes (1) and No (0), excluding missing responses. This variable captures whether respondents used telemedicine or virtual care services for medical purposes within the past year. (2) Gender (SEX_A) categorized as Male and Female. (3) Age group (AGE65) recoded as <65 years and ≥65 years. (4) Race/ethnicity (HISPALLP_A) recoded into six categories: Hispanic, Non-Hispanic (NH) White only, NH Black only, NH Asian only, NH American Indian/Alaska Native (AIAN) only, and Other (including multiracial and other race combinations). (5) Education level (EDUCP_A) collapsed into three categories: High school or less (no degree, GED, high school graduate), Some college/associate degree, and Bachelor’s degree or higher. (6) General health status (PHSTAT_A) was used as a five-level categorical variable: Excellent, Very Good, Good, Fair, and Poor. (7) Geographic region (REGION) as defined by the U.S. Census Bureau: Northeast, Midwest, South, and West. (8) Family Income-to-Poverty Ratio (RATCAT_A) was grouped into four categories: Below Poverty (<1.0); Near Poor/Low Income (1.0–2.99); Moderate Income (3.0–4.99); High Income (≥5.0). Responses labeled “Not Ascertained” were excluded. (9) Health insurance status (NOTCOV_A), recoded as insured versus uninsured. (10) Dental coverage variables, including single-service dental coverage (SINCOVDE_A), Medicare Advantage dental coverage (MCDNCOV_A), and private plan dental coverage (PRDNCOV1_A and PRDNCOV2_A), were combined into a composite indicator of any dental coverage, defined as Yes if any of these sources covered dental care and No otherwise. (11) Urban–rural residence (URBRRL), recoded according to the 2013 NCHS Urban–Rural Classification Scheme as urban (categories 1–3) and rural (category 4). Responses labeled “Refused,” “Not Ascertained,” or “Don’t Know” were excluded from the analytic sample.

### 2.3. Statistical Analysis Plan

All analyses were performed using R version 4.5.1 within the RStudio IDE (version 2025.09.0+387). Descriptive statistics were calculated using survey-weighted estimates to account for the complex sampling design. Frequencies and weighted percentages were reported for categorical variables stratified by delay in dental care. Associations between independent variables and the outcome were first assessed using chi-square tests. Univariable logistic regression models were fitted to estimate crude odds ratios (ORs) and 95% confidence intervals (CIs) for each covariate. These analyses were conducted to describe unadjusted associations and are presented in Supplementary Table S1. All conceptually relevant variables, identified a priori based on theoretical frameworks (including the Andersen Behavioral Model of healthcare utilization) and the prior literature, were initially included in the multivariable model. Model refinement was guided by assessment of statistical contribution, collinearity diagnostics, and overall model fit, while preserving theoretical coherence. Variables that did not meaningfully improve model fit were excluded to maintain parsimony. Subsequently, multivariable logistic regression models were fitted to estimate adjusted odds ratios (AORs) and 95% confidence intervals (CIs) for predictors of cost-related delay in dental care. All models incorporated NHIS weights, strata, and clustering to produce nationally representative and design-corrected estimates. Statistical significance was set at *p* < 0.05 (two-sided). Data visualization and model diagnostics were also conducted to ensure robust model performance and interpretability.

## 3. Results

Table 1 presents the demographic and socioeconomic characteristics of respondents stratified by whether they reported a delay in dental care due to cost in the past 12 months. Dental care utilization differed markedly by delay status. Adults who had not visited a dentist within the past 12 months were substantially more likely to report cost-related delays (36.5%) compared with those who had seen a dental provider in the past year (20.0%) (*p* < 0.001). Telemedicine use was also associated with delay status. Among individuals who used telemedicine in the past 12 months, 33.0% reported delaying dental care due to cost, compared with 27.5% among those who did not use telemedicine (*p* = 0.0219). Women were more likely than men to report delaying dental care due to cost (31% vs. 27%, *p* < 0.001). Cost-related delays were also more common among adults younger than 65 years, with 32% reporting delays compared with 19% among adults aged 65 years and older (*p* < 0.001). Racial and ethnic differences were evident (*p* < 0.001). The prevalence of cost-related delays was highest among Hispanic adults (36%), whereas non-Hispanic Asian adults reported lower prevalence (20%). In contrast, self-rated health status showed a strong gradient (*p* < 0.001). Adults reporting fair or poor health were more likely to delay dental care due to cost compared with those reporting excellent or very good health. Geographic variation was also observed (*p* < 0.001). The prevalence of delay was highest in the South (35%), while the Northeast had the lowest prevalence (17%). In contrast, education level and family income-to-poverty ratio categories were not significantly associated with cost-related dental care delays in this sample. The full analytic sample size summary is presented in Supplementary Table S2.

Table 2 shows the results of the multivariable logistic regression models predicting the odds of delaying dental care due to cost in the past 12 months among U.S. adults. Individuals who used telemedicine services during the past year had higher odds of reporting cost-related delay in dental care (AOR = 1.38; 95% CI: 1.14, 1.67) than those who did not use telemedicine. Women had significantly higher odds of delaying dental care due to cost (AOR = 1.33; 95% CI: 1.10, 1.61) compared with men. Age was strongly associated with the outcome, with adults aged 65 years and older exhibiting substantially lower odds of cost-related dental care delay (AOR = 0.55; 95% CI: 0.45, 0.67) relative to adults younger than 65 years. Individuals identifying as non-Hispanic American Indian or Alaska Native had lower odds of delay (AOR = 0.30; 95% CI: 0.13, 0.67) relative to non-Hispanic White adults. However, this estimate should be interpreted with caution due to the relatively small sample size of this subgroup. Compared with adults reporting excellent health, those reporting good health (AOR = 1.75; 95% CI: 1.31, 2.35), fair health (AOR = 2.40; 95% CI: 1.79, 3.23), and poor health (AOR = 2.26; 95% CI: 1.56, 3.26) had significantly greater odds of delaying dental care due to cost. Compared with adults residing in the Northeast, those living in the Midwest (AOR = 1.50; 95% CI: 1.11, 2.03), South (AOR = 2.64; 95% CI: 2.02, 3.47), and West (AOR = 2.14; 95% CI: 1.57, 2.90) had higher odds of reporting delays in dental care due to cost. Uninsured individuals had substantially higher odds of delay compared with insured individuals (AOR = 2.96; 95% CI: 2.31, 3.79). In contrast, individuals with any form of dental coverage had lower odds of delay (AOR = 0.68; 95% CI: 0.56, 0.83) compared with those without dental coverage. Residence in rural areas was associated with lower odds of delay compared with urban residence (AOR = 0.77; 95% CI: 0.61, 0.96). A sensitivity multivariable logistic regression model that additionally adjusted for dental care utilization in the past 12 months is presented in Supplementary Table S3, and model fit diagnostics for both models are reported in Table S4. The sensitivity model demonstrated improved model fit compared with the primary model, as indicated by lower AIC and deviance values and higher pseudo-R^2^. However, because dental care utilization overlaps conceptually and temporally with the outcome of cost-related delay, it was excluded from the primary model to avoid potential overadjustment and circularity. The consistency of results across both models supports the robustness of the observed associations.

## 4. Discussion

The study found that cost-related delays in dental care remain common among U.S. adults and are strongly patterned by healthcare engagement and sociodemographic characteristics. Telemedicine users in the past 12 months showed higher odds of cost-related dental care delay (AOR = 1.38, 95% CI: 1.14–1.67, *p* = 0.004). The cross-sectional study design prevents causal interference; association is likely confounded by health needs, healthcare engagement patterns, or socioeconomic factors rather than a direct effect of telemedicine on dental access. Disparities were evident across gender, age, race and ethnicity, health status, and geographic region. These findings highlight persistent structural barriers to access to dental care in the United States. These findings suggest that engagement with digital health services does not necessarily translate into improved access to oral health services.

These findings suggest that utilization of digital health services does not necessarily translate into better healthcare access. Individuals who regularly interact with dental providers may benefit from established care relationships, preventive counseling, and earlier identification of oral health needs [22]. Prior research consistently shows that regular dental visits are associated with better oral health outcomes and reduced emergency dental care utilization [1,3]. At the same time, individuals who lack access to routine dental care may delay treatment until symptoms become severe or financially unaffordable. This pattern emphasizes the importance of sustained engagement with preventive oral health services as a pathway to reducing downstream disparities in oral health outcomes.

One of our central findings is the observed association between telemedicine use and higher odds of cost-related dental care delay. Although this relationship may seem counterintuitive, telemedicine use may reflect broader patterns of unmet healthcare needs in the community or issues with healthcare system engagement rather than improved access to dental services specifically [23]. Previous research has shown that telehealth adoption is strongly associated with demographic and socioeconomic characteristics, including digital access, health literacy, and prior healthcare engagement [8,16]. These factors may simultaneously influence both telehealth use and dental care utilization patterns. The NHIS variable used in this study captures general medical care delivery rather than dental-specific services. Although emerging models demonstrate the feasibility of teledentistry for screening, triage, and follow-up care, implementation remains limited across many healthcare systems and was not measured in this study [10,20]. As a result, individuals may engage with telehealth services for medical concerns while still facing persistent financial or structural barriers to obtaining dental care. This finding highlights the potential disconnect between broader telehealth expansion and the integration of oral health services within digital care delivery models.

The sociodemographic disparities observed in this study are consistent with longstanding inequities in access to oral healthcare in the United States. Hispanic adults experienced higher odds of cost-related dental care delay, which aligns with prior evidence showing that minority populations often face structural barriers to preventive dental services [1,4]. Differences by age were also evident, with adults aged 65 years and older reporting lower odds of delay. This pattern may reflect greater insurance coverage through Medicare Advantage plans that include dental benefits or differences in healthcare utilization patterns among older adults. Regional disparities were also notable, with higher odds of delay observed among residents of the South and West compared with those living in the Northeast. Geographic differences in dental workforce distribution, insurance coverage patterns, and state-level health policy environments may contribute to these regional variations in access to care [24]. However, our results showed that income was not significantly associated with delay in the adjusted analysis, suggesting that broader financial and access-related factors beyond income alone may drive cost-related barriers to dental care. Some plausible explanations include the inability of the income-to-poverty ratio categories in NHIS to fully capture financial strain or out-of-pocket affordability for dental services. Further, structural factors such as the availability of dental providers and cost-sharing requirements may play a predominant role rather than income alone. Education was not significantly associated with cost-related dental care delay in the unadjusted analysis. This may suggest that education alone does not fully capture the financial, structural, or insurance-related barriers that shape access to dental care.

This study found that adults with fair or poor self-reported health status had higher odds of experiencing delays in accessing dental services. This finding may reflect the complex interaction between chronic health conditions, healthcare utilization patterns, and financial constraints. Individuals with poorer health often face competing medical expenses that may reduce their ability to prioritize preventive dental care [25]. Previous studies have documented similar associations between overall health status and oral health access, emphasizing the interconnected nature of oral health and general health outcomes [3].

Our study findings have important implications for health policy and healthcare system planning. The results support the broader notion that, rather than fixing downstream disparities after they materialize, addressing upstream inequities in economic status and financial barriers is equally critical to ensuring access to dental care for many adults [26]. Although telehealth expansion has transformed aspects of healthcare delivery, our study findings suggest that digital access alone may be insufficient to reduce cost-related delays in dental care [27]. This distinction should be noted because access to telemedicine does not necessarily indicate integration of oral health services into digital care pathways. Service-integration gaps may help explain why telemedicine engagement does not necessarily correspond with improved dental care access. Those gaps include issues with dental referral mechanisms, dental benefit coverage, and direct oral health linkage during general telemedicine encounters. Policy initiatives aimed at expanding dental coverage and strengthening access to preventive dental care may help reduce cost-related delays in care.

This study has several limitations that should be considered when interpreting the findings. First, the cross-sectional design of the NHIS limits the ability to establish causal relationships between telemedicine use and delayed dental care. So, our associations should not be taken to imply causal relationships. Second, the self-reported survey responses may be subject to recall bias or reporting errors. Third, the NHIS does not capture detailed information on several contextual variables, including dental insurance coverage or the type of telehealth services used, which may influence patterns of care utilization. These unobserved variables may bias the observed associations and limit causal interpretation. Fourth, the NHIS does not capture information on the source of dental care, which may result in residual confounding. Moreover, the primary exposure variable (VIRAPP12M_A) captures general telemedicine or virtual care use and does not distinguish dental telehealth (teledentistry) encounters. As such, this study cannot directly assess oral health-specific digital care pathways, which limits the interpretation of findings related to telehealth and dental care access. In addition, the conceptual and temporal overlap between recent dental visits and the outcome of cost-related delay may introduce interpretive ambiguity, although we addressed this through sensitivity analyses. Further, estimates for smaller subgroups, such as non-Hispanic American Indian or Alaska Native populations, may be unstable and should be interpreted with caution. Lastly, although the NHIS provides nationally representative data, certain population subgroups may be underrepresented in household surveys. Future research should further examine how digital health engagement intersects with access to oral healthcare. Longitudinal and mixed methods studies may help clarify whether telehealth use influences subsequent dental care utilization or referral pathways. Additional research is also needed to evaluate how teledentistry models can be integrated into primary care, community health programs, and public health systems to improve access for underserved populations. Understanding how digital health infrastructure can support preventive oral health services will be increasingly important as healthcare delivery continues to evolve.

## 5. Conclusions

This study examined national patterns of cost-related delays in dental care among U.S. adults using 2023 NHIS data. Our results indicate that such delays remain common and are strongly associated with patterns of healthcare engagement and sociodemographic characteristics. Individuals who had not visited a dental provider in the past year were substantially more likely to report delaying dental care due to cost. Telemedicine users also had higher odds of experiencing delays, though this association does not establish whether digital engagement with the healthcare system improves access to oral health services. The observed differences across gender, race and ethnicity, health status, and geographic region signal persistent structural inequities in oral health access across the United States. These findings highlight the need for policy approaches that address financial barriers to dental care while also strengthening the integration of oral health within broader digital health and healthcare delivery systems. Expanding coverage for preventive dental services, improving digital literacy, and developing referral pathways that connect telehealth encounters with dental care services may help reduce cost-related delays and improve equitable access to oral healthcare.

## Figures and Tables

**Table 1:** Demographic characteristics by dental care delay status.

Variable	Level	Delay in Dental Care Due to Cost, Past 12 Months *N* ^1^ (%) ^2^	No Delay in Dental Care Due to Cost, Past 12 Months *N* ^1^ (%) ^2^	*p*-Value
Visited dental provider in past 12 months	No	7940 (36.5%)	13,800 (63.5%)	<0.001
	Yes	3920 (20%)	15,350 (80%)	
General telemedicine use in past 12 months	No	8380 (27.5%)	22,130 (72.5%)	0.0219
	Yes	3480 (33%)	7020 (67%)	
Gender	Male	4210 (27%)	11,530 (73%)	0.0000
	Female	7650 (31%)	17,620 (69%)	
Age group (years)	<65	9190 (32%)	18,180 (68%)	<0.001
	65+	2670 (19%)	10,970 (81%)	
Race/ethnicity	Hispanic	3710 (36%)	6560 (64%)	<0.001
	NH White only	5620 (27%)	14,660 (73%)	
	NH Black only	1850 (27%)	5510 (73%)	
	NH Asian only	340 (20%)	1310 (80%)	
	NH AIAN only	90 (14%)	510 (86%)	
	Other	250 (25%)	600 (75%)	
Education level	HS or less	9570 (29%)	23,870 (71%)	0.6285
	Some college/AD	2190 (31%)	4960 (69%)	
	Bachelor and higher	100 (23%)	320 (77%)	
Self-assessment of health	Excellent	1450 (24%)	4510 (76%)	<0.001
	Very good	2220 (25%)	6380 (75%)	
	Good	3800 (32%)	8870 (68%)	
	Fair	3190 (34%)	6570 (66%)	
	Poor	1200 (31%)	2820 (69%)	
U.S. Census region	Northeast	970 (17%)	4530 (83%)	<0.001
	Midwest	1790 (23%)	6210 (77%)	
	South	6070 (35%)	11,830 (65%)	
	West	3030 (31%)	6580 (69%)	
Family Income-to-Poverty Ratio	Below Poverty	2350 (29%)	5880 (71%)	0.9548
	Near Poor/Low Income	2560 (29%)	6430 (71%)	
	Middle Income	3580 (29%)	9070 (71%)	
	High Income	3370 (30%)	7770 (70%)	
Health insurance status	Insured	9040 (25%)	26,650 (75%)	<0.001
	Uninsured	2950 (53%)	2560 (47%)	
Any dental coverage	Yes	2620 (21%)	10,020 (79%)	<0.001
Urban/rural status	Urban	9710 (31%)	22,280 (69%)	0.006
	Rural	2280 (25%)	6930 (75%)	

Abbreviations: AD—associate degree; AIAN—American Indian or Alaska Native; HS—high school; NH—Non-Hispanic. ^1^ unweighted; ^2^ weighted.

**Table 2:** Multivariable regression predicting delay in dental care due to cost in the past 12 months, NHIS 2023.

Variable	Level	AOR	CI	*p*-Value
General telemedicine use in past 12 months	No	1.00		
	Yes	1.38	1.14, 1.67	0.001
Gender	Female	1.33	1.10, 1.61	0.004
	Male	1.00		
Age group (years)	<65	1.00		
	65+	0.55	0.45, 0.67	<0.001
Race/ethnicity	NH White only	1.00		
	Hispanic	1.09	0.87, 1.36	0.5
	NH Black only	0.87	0.68, 1.11	0.3
	NH Asian only	0.77	0.49, 1.20	0.2
	NH AIAN only	0.30	0.13, 0.67	0.004
	Other	0.58	0.31, 1.08	0.088
Self-assessment of health	Excellent	-	-	
	Very good	1.29	0.95, 1.75	0.11
	Good	1.75	1.31, 2.35	<0.001
	Fair	2.40	1.79, 3.23	<0.001
	Poor	2.26	1.56, 3.26	<0.001
U.S. Census region	Northeast	1.00		
	Midwest	1.50	1.11,2.03	0.009
	South	2.64	2.02, 3.47	<0.001
	West	2.14	1.57, 2.90	<0.001
Health insurance status	Insured	1.00		
	Uninsured	2.96	2.31, 3.79	<0.001
Any dental coverage	No	1.00		
	Yes	0.68	0.56, 0.83	<0.001
Urban/rural status	Urban	1.00		
	Rural	0.77	0.61, 0.96	0.022

Abbreviations: AIAN—American Indian or Alaska Native; AOR—adjusted odds ratio; CI—confidence interval; NH—Non-Hispanic.

## Data Availability

The data analyzed in this study are publicly available from the National Health Interview Survey (NHIS), conducted by the National Center for Health Statistics (NCHS), Centers for Disease Control and Prevention. The dataset used for this study (NHIS 2023) is available at https://www.cdc.gov/nchs/nhis/index.htm (accessed on 13 December 2025).

## Supplementary Materials

The following supporting information can be downloaded at https://www.mdpi.com/article/10.3390/clinpract16050091/s1, Table S1: Univariable Regression Predicting Delay in Dental Care Due to Cost in the Past 12 Months, NHIS 2023. Table S2. Sample size summary. Table S3. Sensitivity analysis additionally adjusting for dental visit in the past 12 months. Table S4. Model fit summary.

## Abbreviations

The following abbreviations are used in this manuscript:

AD	Associate Degree
AIAN	American Indian or Alaska Native
AOR	Adjusted Odds Ratio
CDC	Centers for Disease Control and Prevention
CI	Confidence Interval
NCHS	National Center for Health Statistics
NH	Non-Hispanic
NHIS	National Health Interview Survey
OR	Odds Ratio
TMD	Temporomandibular Disorders
T-Med	Telemedicine for Temporomandibular Disorder Management
