# Supplementary material for: Digital Access for Screens and Smiles: Telehealth Use and Dental Care Access in the U.S
Source: Clin Pract. 2026 May 6;16(5):91. doi: 10.3390/clinpract16050091 (PMC13204784; doi:10.3390/clinpract16050091)
Supplement: Supplementary file 1 [file clinpract-16-00091-s001.zip › clinpract-4273310-supplementary.pdf]

Table S1. Univariable Regression Predicting Delay in Dental Care Due to Cost in the Past 12 Months, NHIS 2023.

Variable	Level	OR	CI	p-value
Visited dental provider in past 12 months	Yes	0.41	0.34, 0.48	<0.001
	No	1.00		
General telemedicine use in past 12 months	No	1.00		
	Yes	1.24	[1.03, 1.49]	0.022
Gender	Female	1.25	[1.05, 1.50]	0.013
	Male	1.00		
Age group (years)	<65	1.00		
	65+	0.48	[0.40, 0.58]	<0.001
Race/ethnicity	NH White only	1.00		
	Hispanic	1.55	1.27, 1.89	<0.001
	NH Black only	1.03	0.82, 1.30	0.8
	NH Asian only	0.68	0.43, 1.08	0.10
	NH AIAN only	0.43	0.19, 1.00	0.049
	Other	0.89	0.49, 1.65	0.7
Education level	Bachelor+	0.74	[0.30, 1.83]	0.5
	Some college/AD	1.08	[0.87, 1.34]	0.5
	HS or less	1.00		
Self-assessment of health	Excellent	1.00		
	Very good	1.10	[0.83, 1.46]	0.5
	Good	1.51	[1.14, 1.99]	0.004
	Fair	1.70	[1.30, 2.22]	<0.001
	Poor	1.45	[1.03, 2.04]	0.032
U.S. Census region	Northeast	1.00		
	Midwest	1.40	[1.04, 1.88]	0.026
	South	2.56	[1.97, 3.34]	<0.001
	West	2.14	[1.59, 2.89]	<0.001
Family Income-to-Poverty Ratio	High Income	1.02	0.80, 1.31	0.8
	Moderate Income	0.98	0.77, 1.24	0.8
	Near Poor/Low Income	0.96	0.75, 1.24	0.8
	Below Poverty	1.00		
Health insurance status	Insured	1.00		
	Uninsured	3.43	2.74, 4.28	<0.001
Any dental coverage	No	1.00		
	Yes	0.52	0.43, 0.63	<0.001
Urban/rural status	Urban	1.00		
	Rural	0.73	0.59, 0.91	0.006

Abbreviations: AD – associate degree; AIAN - American Indian or Alaska Native; AOR – adjusted odds ratio; CI – confidence interval; HS – high school; NH – Non-Hispanic; OR – odds ratio.

Table S2. Sample size summary.

Item	Value
Main analytic unweighted N	41,200

Item	Value
Sensitivity analytic unweighted N	40,480
Weighted N, main analytic sample	343,760,489
Weighted N, sensitivity analytic sample	336,301,287

Table S3. Sensitivity analysis additionally adjusting for dental visit in the past 12 months.

Characteristic	Adjusted OR	95% CI	p-value
**General telemedicine use in past 12 months**			
No	—	—	
Yes	1.43	1.17, 1.74	<0.001
**Visited dental provider in past 12 months**			
No	—	—	
Yes	0.42	0.35, 0.51	<0.001
**Sex**			
Male	—	—	
Female	1.39	1.15, 1.68	<0.001
**Age group**			
<65 years	—	—	
≥65 years	0.51	0.42, 0.63	<0.001
**Race/ethnicity**			
NH White only	—	—	
Hispanic	1.20	0.96, 1.51	0.11
NH Black only	0.93	0.72, 1.19	0.6
NH Asian only	0.77	0.47, 1.26	0.3
NH AIAN only	0.32	0.14, 0.76	0.010
Other	0.61	0.31, 1.21	0.2
**Education level**			
High school or less	—	—	
Some college/associate	1.38	1.08, 1.77	0.010
Bachelor's or higher	1.21	0.48, 3.05	0.7
**Self-rated health**			
Excellent	—	—	
Very good	1.28	0.94, 1.76	0.12
Good	1.70	1.25, 2.32	<0.001
Fair	2.16	1.58, 2.96	<0.001
Poor	2.08	1.41, 3.08	<0.001
**U.S. Census region**			
Northeast	—	—	
Midwest	1.37	1.02, 1.83	0.038

Characteristic	Adjusted OR	95% CI	p-value
South	2.44	1.87, 3.19	<0.001
West	2.13	1.57, 2.91	<0.001
**Family income-to-poverty ratio**			
Below poverty (<1.0)	—	—	
Near poor/low income (1.0–2.99)	0.91	0.70, 1.19	0.5
Moderate income (3.0–4.99)	0.93	0.72, 1.20	0.6
High income (≥5.0)	0.99	0.77, 1.28	>0.9
**Health insurance status**			
Insured	—	—	
Uninsured	2.69	2.07, 3.49	<0.001
**Any dental coverage**			
No	—	—	
Yes	0.77	0.63, 0.95	0.014
**Urban/rural status**			
Urban	—	—	
Rural	0.72	0.57, 0.91	0.005

Abbreviations: CI = Confidence Interval, OR = Odds Ratio

Table S4. Model fit summary.

Model	N	AIC	Deviance	Null deviance	Residual df	Pseudo R^2 McFadden
Main model	41,200	46,135.22746	45,496.49	50,135.17	564	0.09252342
Sensitivity model	40,480	44,107.82556	43,438.38	49,151.11	563	0.11622782
